# Utility of sFtl-1 and Placental Growth Factor Ratio for Adequate Preeclampsia Management

**DOI:** 10.3390/healthcare11030381

**Published:** 2023-01-29

**Authors:** Elena Ciciu, Ana-Maria Paṣatu-Cornea, Stefania Dumitru, Lucian Cristian Petcu, Camer Salim, Liliana-Ana Tuta

**Affiliations:** 1 Nephrology Department, Constanta County Emergency Hospital, 900591 Constanţa, Romania; 2 Department of Clinical Medical Sciences, Faculty of General Medicine, Ovidius University of Constanţa, 900527 Constanţa, Romania; 3 Department of Biophysics and Biostatics, Faculty of Dental Medicine, Ovidius University of Constanţa, 900527 Constanţa, Romania; 4 Emergency Department, Constanta County Emergency Hospital, 900601 Constanta, Romania; 5 Doctoral School, Faculty of Medicine, “Ovidius” University of Constanţa, 900527 Constanţa, Romania

**Keywords:** pregnancy, preeclampsia, gestational hypertension, biomarkers, sFtl-1/PIGF ratio

## Abstract

**Introduction:** The pathophysiology of preeclampsia is represented by placental ischemia and the release of angiogenic factors. Recent research suggests that using the value of the sFtl-1/PIGF ratio is more accurate for monitoring angiogenic activity. The aim of this study consists in assessing the clinical utility of the sFtl-1/PIGF ratio in determining the diagnosis and severity of preeclampsia. **Material and Methods:** In our study a descriptive and prospective plan was used for analyzing the specific value of the sFtl-1/PIGF ratio in women with preeclampsia and in women with gestational hypertension, depending on the gestational age and severity. **Results:** The study included 59 women with preeclampsia and 25 women with gestational hypertension. The mean value of the sFtl-1/PIGF ratio of pregnant women with preeclampsia was 209.2 pg/mL, while in the gestational hypertension group, the mean value of the sFtl-1/PIGF ratio was 46.08 pg/mL. The difference between the value of the sFtl-1/PIGF ratio of the group with preeclampsia and the gestational hypertension group was > 67 (pg/mL), with a sensitivity of 86.44% and a specificity of 92.00%. Significant differences were found between the median values of the sFtl-1/PIGF ratio in pregnant women with severe preeclampsia in the early-onset subgroup compared to those in the late-onset subgroup (307 pg/mL, and 98 pg/mL, respectively, *p* = 0.009 < α = 0.05). **Conclusions:** The sFtl-1/PIGF ratio may be an alternative method for diagnosing preeclampsia and it can provide data about this condition’s severity.

## 1. Introduction

The morpho-pathological change that is the basis of preeclampsia (PE) is the trophoblastic invasion with remodeling of the maternal health. Thus, an imbalance is triggered between the regulatory factors of the placental secretion responsible for the angiogenesis of the maternal circulation, which leads to low concentrations of proangiogenic factors (PIGF, VEGF) and increased concentrations of antiangiogenic factors (sFtl-1, sEng) [1,2]. Studies suggest that these angiogenic biomarkers may be associated with the severity of PE and also may be considered as serological biomarkers in the diagnosis of PE [3,4,5]. Recent research suggests that using the value of the sFtl-1/PIGF ratio would be much more accurate for determining the severity and diagnosis of PE [6,7]. However, the values of these biomarkers and of the ratio were not similar in existing studies, depending on the epidemiology of each country [8,9,10,11,12,13,14].

In Romania, the diagnosis of PE is based on clinical signs (the presence of high blood pressure values) and non-specific biological values (24 h urine protein test), which cannot be used in the early diagnosis and adequate management of this condition [15]. Romania is facing one of the highest maternal and neonatal mortality and morbidity rates in Europe. Recently, collected data showed that, at the European Union level, 12.24% of the yearly maternal mortalities were registered in Romania, and approximately 10% of these came from the Dobrogea region, southeastern Romania [16]. Using a biomarker with high sensitivity and specificity could lead to an early diagnosis of the disease and the management of its complications, reducing maternal and perinatal mortality in our region [4,17].

## 2. Material and Methods

In our study a prospective and descriptive plan was used, based on a group of 59 pregnant women with PE who were compared with 2 non-PE groups: one with gestational hypertension, GH, including 25 pregnant women, and the control group, CG, including 43 pregnant women. The study was performed between May 2017 and May 2021 and included pregnant women treated at the main emergency hospital in southeastern Romania, the Constanta County Emergency Clinical Hospital. All the patients were hospitalized in the Gynecology ward and were interdisciplinarily monitored by the Nephrology Department team, with regular follow-up visits.

All the pregnant women with a gestational age greater than 20 weeks who fulfilled the PE diagnosis criteria were included in the study. Pregnant women with a history of chronic diseases such as essential (primary) hypertension, diabetes, and chronic nephropathy, and pregnant women under the age of 16, were excluded from the study. The clinical variables surveyed were maternal age, gestational age at onset, and severity. The diagnosis of PE and GH was established according to the diagnosis recommendations of the Romanian Society of Obstetrics and Gynecology approved by the Ministry of Health under order no. 1.241/9 August 2019 [15]. Severe PE was defined as including the following criteria: persistent and severe hypertension, renal failure, thrombocytopenia, hepatocytolysis, eclampsia, persistent headache, and visual disturbances. Depending on the onset of the pathology, the study groups were divided into two subgroups: early-onset (before 34 weeks of pregnancy) and late-onset (after 34 weeks of pregnancy).

To complete the biological profile of the patients included in the study, the following variables were determined: proteinuria/24 h, PE biomarkers (sFtl1, PIGF, sFtl-1/IPGF ratio), uric acid, serum creatinine, complete blood count (especially platelet count), and liver enzymes (ALT, AST). For the determination of the PE biomarkers (sFtl1, PIGF, sFtl-1/PIGF ratio) a minimum of 1 mL of venous blood was collected in a vacutainer without anticoagulant, centrifuged, and processed in a maximum of 3 h. If the processing of the sample was not possible in the 3 h time limit, the serum was kept for 8 h at a temperature between 2 and 8 °C and subsequently frozen at −20 °C so as to remain stable under these conditions for a period of one month. The processing of the samples was performed using Elecsys automated assays on an immunochemical platform with electrochemiluminescence detection (ECLIA) [18].

The experimental data were processed using IBM SPSS Statistics 23 and MedCalc 14.8.1. The procedures used were descriptive statistics, graphs, parametric statistical tests, non-parametric statistical tests for categorical variables, non-parametric statistical tests for ordinal data or for numerical variables when the normal condition was not satisfied, and ROC curve analysis.

## 3. Results

### 3.1. Clinical and Paraclinical Features of the Studied Groups (PE, GH, Control Group)

The study included 127 pregnant women: 59 (46.46%) pregnant women with PE, 25 (19.69%) pregnant women with GH, and 43 (33.86%) pregnant women in the control group. The clinical and paraclinical findings are described in Table 1 and Table 2.

### 3.2. Utility of the sFtl-1/PIGF Ratio in the Diagnosis of Preeclampsia

The mean value of the sFtl-1/PIGF ratio of the pregnant women with PE (no. = 59) was 209.2 pg/mL with a standard deviation of 138.77 pg/mL, a median value of 251 pg/mL, and an IQR (P_75_–P_25_) of 229 pg/mL; in the GH group (no. = 25), the mean value of the sFtl-1/PIGF ratio was 46.08 pg/mL, with a standard deviation of 17.37 pg/mL, a median value of 43 pg/mL, and a P_75_–P_25_ of 29.5 pg/mL; lastly, in the control group (no. = 43), the mean value of the sFtl-1/PIGF ratio was 3.9 pg/mL with a standard deviation of 0.20 pg/mL, a median value of 3.90 mg/mL, and a P_75_–P_25_ of 0.30 pg/mL (Table 3).

There were statistically significant differences between the median values of the sFtl-1/PIGF ratio in the three study groups (*p* < 0.001 < α = 0.05, median test), and the distribution of the sFtl-1/PIGF ratio values was different in the analyzed groups (*p* < 0.001 < α = 0.05, Kruskal–Wallis test for independent variables).

### 3.3. ROC Curve of the sFlt-1/PIGF Ratio in the Diagnosis of PE

**Figure 1:**
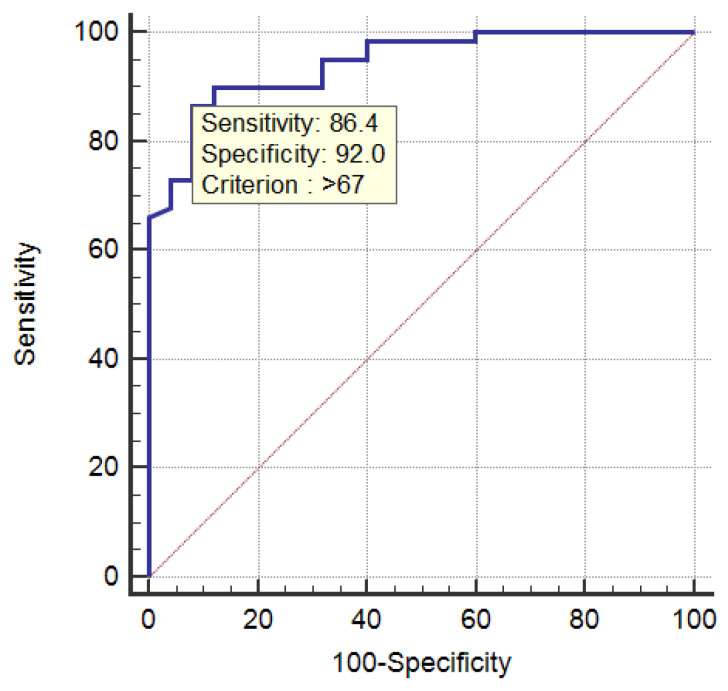
ROC curve of the sFtl-1/PIGF ratio in the diagnosis of PE.

The area under the ROC curve (AUC) was estimated at 0.943 with a confidence interval of 95% between 0.870 and 0.982. The statistical value of the test was z-score = 18.843 and *p* value < 0.0001 < α = 0.05, which is why the area under the curve was different from 0.5. The Youden index was 0.7844, and the difference in the value of the sFtl-1/PIGF ratio between the group with PE and the GH group was >67 (pg/mL), with a sensitivity of 86.44% and a specificity of 92.00% (Figure 1).

### 3.4. The Value of the sFtl-1/PIGF Ratio According to Gestational Age at the Onset of Preeclampsia

Depending on the gestational age at the onset of PE, the study groups were divided into two subgroups, early-onset (<34 weeks) and late-onset (>34 weeks), and the number of pregnant women in each group is described in Table 4.

### 3.5. The Value of the sFtl-1/PIGF Ratio According to Gestational Age at the Onset of Preeclampsia

There were statistically significant differences in the median values of the sFtl-1/PIGF ratio among all the subgroups of women with early onset (*p* < 0.001 < α = 0.05, median test).

In addition, there were statistically significant differences in the median values of the sFtl-1/PIGF ratio in the three subgroups of pregnant women with late onset (*p* < 0.001 < α = 0.05, median test). A pairwise comparison test revealed significant differences between the pregnant women with late-onset GH and late-onset PE compared to the control group (*p* < 0.001), but there was no significant difference between the subgroup of pregnant women with late-onset PE compared with those with late-onset GH (*p* = 0.102 > 0.05), as in shown in Table 5.

### 3.6. The Value of the sFtl-1/PIGF Ratio According to Gestational Age at the Onset and the Severity of Preeclampsia

Depending on the severity of PE, the study group included 23 (38.98%) pregnant women with mild PE and 36 (61.02%) pregnant women with severe PE.

The median value of the sFtl-1/PIGF ratio in the group with mild PE (no. = 23) was 77 pg/mL with a minimum of 39 pg/mL and a maximum of 98 pg/mL; for severe PE (no. = 36) the median value of the sFtl-1/PIGF ratio was 303 pg/mL, with a minimum of 49 pg/mL and a maximum of 424 pg/mL (Table 6).

**Figure 2:**
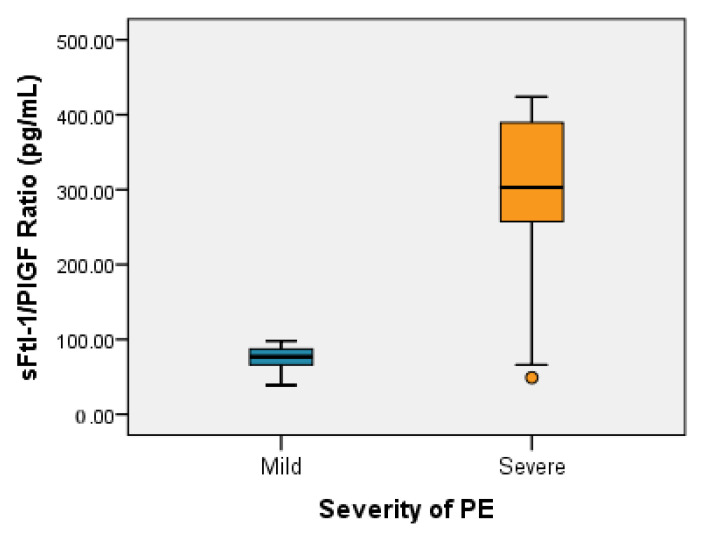
Box–whisker plots representing sFtl1-/PIGF ratio values depending on PE severity.

There were statistically significant differences between the median values of the sFtl-1/PIGF ratio in the group with mild versus severe impairment (*p* < 0.001 < α = 0.05, median test) as shown in Figure 2.

### 3.7. ROC Curve of sFlt-1/PIGF Ratio in the Severity of PE

**Figure 3:**
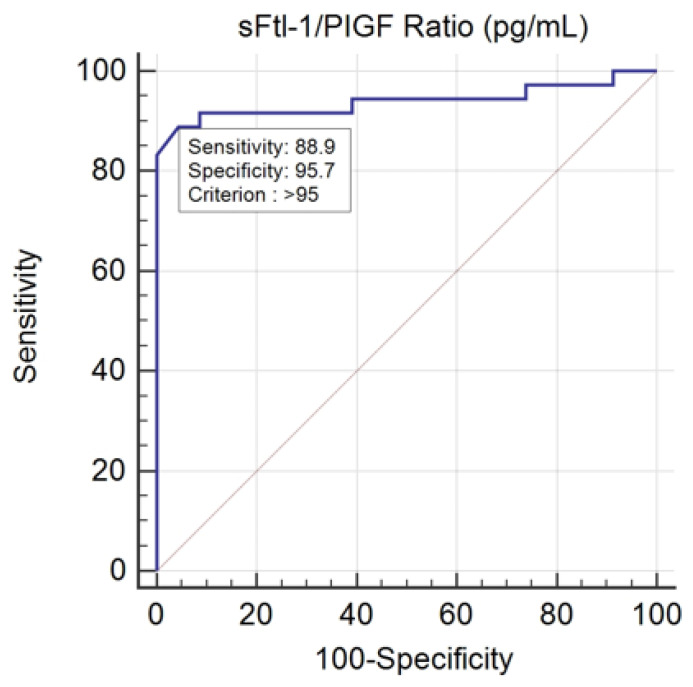
ROC curve of sFtl-1/PIGF ratio in mild versus severe PE.

The area under the ROC curve (AUC) was estimated at 0.940 with a confidence interval of 95% between 0.845 and 0.985. The statistical value of the test was z-score = 12,869 and *p*-value < 0.0001 < α = 0.05, which is why the area under the curve was different from 0.5. The Youden index was 0.8454 and the difference between the value of the sFtl-1/PIGF ratio in the severe versus mild PE subgroups was >95 (pg/mL), with a sensitivity of 88.89% and a specificity of 95.65%, as shown in Figure 3.

### 3.8. The Value of the sFtl-1/PIGF Ratio According to Gestational Age at Onset and Severity of Preeclampsia

**Figure 4:**
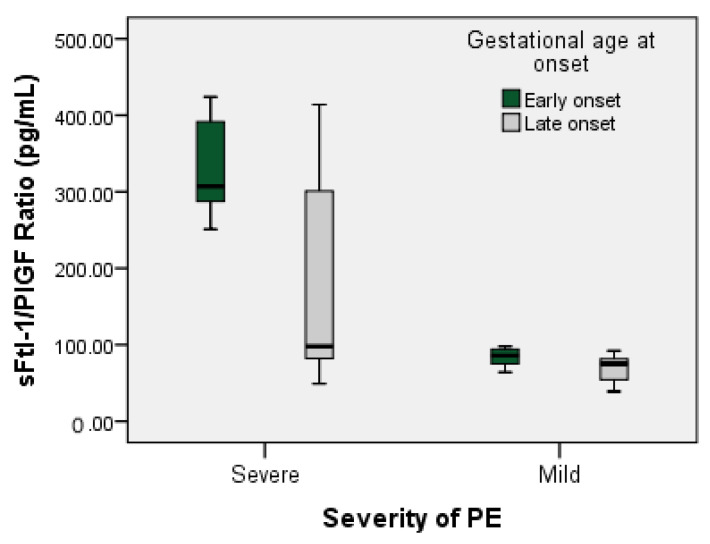
Box–whisker plots representing sFtl-1/PIGF ratio values depending on severity at onset of PE.

The values of sFtl-1/PIGF according to the severity and onset of PE are represented in Table 7 and Figure 4.

The risk of finding severe PE in pregnant women with early onset is 5625 times higher than the risk of finding severe PE in pregnant women with late onset (OR = 5.625, 95% CI for OR = 1.794–17.633).

Pregnant women with early-onset PE (<34 weeks) had a higher sFlt-1/PIGF ratio value in the severe PE subgroup compared to the mild PE subgroup, evincing statistical significance (307.00 pg/mL compared to 86.00 pg/mL, *p* = 0.011 < α = 0.05). Pregnant women with late-onset PE (>34 weeks) had a similar mean value of sFlt-1/PIGF ratio in the severe PE and mild PE subgroups, but statistical significance was evident (98.00 pg/mL versus 75.00 pg/mL, *p* = 0.033 < α = 0.05).

Significant differences were found between the median values of the sFtl-1/PIGF ratio in pregnant women with severe PE in the early-onset subgroup compared to those in the late-onset subgroup (307.00 pg/mL, respectively 98.00 pg/mL, *p* = 0.009 < α = 0.05), and its distribution evinces statistical significance. In contrast, the median value of the sFtl-1/PIGF ratio in pregnant women with mild PE did not reach statistical significance in the early-onset subgroup compared to the late-onset subgroup (86.00 pg/mL and 75.00 pg/mL, respectively; *p* = 0.400 > α = 0.05).

## 4. Discussion

The study of the pathogenesis of PE has deepened in recent decades, with research suggesting that the pathophysiology is different when comparing early PE (<34 weeks of gestation) to late PE (>34 weeks of gestation) [19,20,21]. PE with onset before the 34^th^ week of pregnancy, also called placental PE, is caused by placental hypoxia associated with oxidative stress. PE with onset after the 34^th^ week of pregnancy, also called maternal PE, occurs because of the interaction between the placenta and the mother’s organism, with associated autoimmune kidney diseases, metabolic diseases, chronic hypertension, and diabetes. Studies suggest that the association between the two etiologies of PE (placental hypoxia and maternal pathology) is common [22,23,24].

Starting with 2003–2004, Maynard and Levine have reported the first evidence that supports the fact that the central role in the pathogenesis of placental hypoxia is played by the factors that influence the angiogenesis of the maternal circulation [5,25,26,27]. Fms-like tyrosine kinase (sFtl-1) is a circulating antagonist for vascular growth factors (VEGF, PIGF), circulating levels in preeclampsia being increased due to placental ischemia several weeks before clinical onset, sometimes starting in the first trimester. PIGF (placental growth factor) is a proangiogenic growth factor from the VEGF (vascular endothelial growth factor) family that stimulates the proliferation of placental vessels, altering vascular permeability and trophoblastic activity. An imbalance of these growth factors leads to impaired permeability and integrity of the vascular wall, the final consequence being the appearance of edema, proteinuria, and intracapillary endotheliosis [25].

The diagnosis of PE is mainly based on clinical manifestations and non-specific biochemical tests. Some pregnant women with certain complications, such as chronic hypertension and autoimmune pathologies, are destined to progress to PE. These pregnant women have similar symptoms to PE, but the management and prognosis are different. Sometimes it is difficult to make the correct differential diagnosis based only on the current diagnosis criteria.

Increased serum levels of sFtl-1 and decreased PIGF levels resulted in an increased sFtl-1/PIGF ratio, which could be detected in the second half of pregnancy in pregnant women with PE. Disorders of angiogenic factors seem to be detectable before the appearance of clinical symptoms in PE, thus allowing the differentiation of pregnant women with PE from pregnant women with associated gestational hypertension, or from normotensive pregnancies [28,29]. Recently, some researchers have suggested the use of sFtl-1/PIGF in the prediction and diagnosis of PE [7,27,30,31]. In our study, the mean values of the sFlt-1/PIGF ratio were higher in the group of pregnant women with PE (209 +/− 138,77 pg/mL) than in the control group (3.9 +/− 0.20 pg/mL) or in the GH group (46.08 +/− 17.37 pg/mL, *p* < 0.001). Statistical significance was identified (*p* < 0.001 < α = 0.05) when comparing the three study groups, confirming that the value of the sFtl-1/PIGF ratio in the PE group was much higher compared to the GH group or to the control group. To determine the performance of the sFtl-1/PIGF ratio in clinical practice, the ROC curve was constructed, which highlighted the fact that a sFlt-1/PIGF ratio level of 67 pg/mL represents the threshold between PE and GH, with a sensitivity of 86.44% and a specificity of 92.00%.

Early-onset PE, defined as onset before 34 weeks, is characterized by defective placental implantation and deficient spiral artery remodeling, leading to intrauterine growth restriction and altered expression of placental proteins (sFtl-1) and growth factors (PIGF) [32,33]. Conversely, late-onset preeclampsia is defined as onset after 34 weeks of pregnancy and is not necessarily defined by placental dysfunction, the angiogenic and antiangiogenic factors being dysregulated to a less dramatic extent [34], such findings being highlighted by the values of the sFlt-1/PIGF ratio in the present study.

Our data showed that early-onset PE had a higher median sFlt-1/PIGF ratio compared to the ratio in late-onset PE (302.9 pg/mL versus 78 pg/mL, *p* < 0.001). In the subgroups of pregnant women with gestational age less than 34 weeks, the median values of the sFlt-1/PIGF ratio in pregnant women in the PE group were significantly higher compared to the GH group and to the control group (*p* < 0.001), including a significant difference between pregnant women in the GH group and in the control group (*p* < 0.001). Regarding the subgroups of pregnant women with gestational age greater than 34 weeks, the median value of the sFtl-1/PIGF ratio in the PE subgroup was also increased (*p* = 0.001), but the difference between the PE and GH subgroups did not reach statistical significance (78.00 pg/mL versus 62.5 pg/mL, *p* = 0.102 > 0.05). This finding is consistent with the study of Noori et al. [35] but slightly different from that of von Dadelszen et al. [36], who found significant differences between GH and PE in given gestational age subgroups. Although the number of pregnant women with GH was limited in our study, we were able to highlight the tendencies in this group. The sFlt-1/PIGF ratio is significantly increased in pregnant women with PE compared to GH or pregnancies without other pathologies.

Depending on the severity criteria, the pregnant women in our study could be divided into two subgroups: severe PE and mild PE. Levine et al. [5] and Buhimschi et al. [37] suggest in their studies that the sFtl-1/PIGF ratio can be used to define the severity of preeclampsia. Analyzing the median values of the sFtl-1/PIGF ratio in the severe PE subgroup in the present study (36/59, 303 pg/mL with a minimum of 49 pg/mL and a maximum of 424 pg/mL) reveals statistical significance, with median values in the mild PE group (23/59, 77 pg/mL with a minimum of 39 pg/mL and a maximum of 98 pg/mL; *p* < 0.001 < α = 0.05), confirming the literature finding that severe PE correlates with higher values of the sFtl-1/PIGF ratio. To determine the performance of the sFlt-1/PIGF ratio in clinical practice from our data, the ROC curve of the sFlt/1/PIGF ratio value was constructed, establishing that a level of 95 pg/mL represents the threshold between severe PE and mild PE (sensitivity of 88.89% and specificity of 95.65%). Rana et al. [8] and Nikuei et al. [38] also claimed that a high value of the sFtl-1/PIGF ratio is associated with severe forms of PE and its early onset, representing the most sensitive and specific biomarker to distinguish PE from normal pregnancies keeping in view severe forms and early onset.

The same characteristic was also found in the data of our study, so from the perspective of gestational age, in pregnant women with early-onset PE (<34 weeks), the sFlt-1/PIGF ratio was higher in those with a severe form compared to those with a mild form, evincing statistical significance (307 pg/mL versus 86 pg/mL, *p* = 0.011 < α = 0.05). Pregnant women with late-onset PE (>34 weeks) had a similar mean sFlt-1/PIGF ratio between severe and mild forms but reached statistical significance (98 pg/mL versus 75 pg/mL, *p* = 0.033 < α = 0.05). There were also significant differences between the median values of the sFlt-1/PIGF ratio in pregnant women with severe early-onset PE compared to those with a late onset (307 pg/mL, respectively 98 pg/mL, *p* = 0.009 < α = 0.05), their distribution showing statistical significance. On the other hand, in pregnant women with mild PE, statistical significance was not revealed in terms of the median value of the sFtl-1/PIGF ratio in the subgroups with early versus late onset (86 pg/mL, respectively 75 pg/mL, *p* = 0.400 > α = 0.05). Data from the literature show that the prediction accuracy of the sFtl-1/PIGF ratio was more accurate in pregnant women of gestational age under 34 weeks [39,40]. Since after 36 weeks of pregnancy the sFtl-1/PIGF ratio evinces a general tendency to be higher in all pregnant women, it seems that it would not have the same predictive value at a late gestational age [4,26,27,41].

In Romania, the analysis of maternal mortality, considered one of the most important indicators of the quality of life, is carried out by monitoring four major causes: abortion, direct obstetric risk, indirect obstetric risk, and collateral causes. Comparing the data released by the National Center for Public Health Statistics of Romania, during our study’s time interval (2017–2021), maternal mortality per 100,000 births caused by indirect obstetric risk rose from 4.1% in 2017 to 6.7% in 2021. Furthermore, direct causes of maternal mortality (such as abortion, proteinuria, and hypertension) surged dramatically in the given time frame, from 4.2% in 2017 to 7% in 2020. Maternal mortality, strictly reported in relation to edema, proteinuria, and hypertension, was directly proportional to previously described reports; thus, there was an increase from 1.04% in 2017 to 1.1% in 2019 [16].

Due to these concerning data, it is necessary to increase awareness about renal disorders during pregnancy, to promptly establish a positive diagnosis, and to make appropriate therapeutic decisions. Preeclampsia is associated with a high risk of evolution burdened with complications, and the use of the sFtl-1/PIGF ratio could contribute to an objective follow-up by the nephrological and gynecological team for the optimal completion of the pregnancy and to reduce the risks of both fetal and maternal mortality.

Since this is the first study describing the utility of sFtl-1/PIGF ratio in the diagnosis and severity assessment of preeclampsia, which is directly related to maternal morbidity and mortality in our region, we presume that the results of our research regarding particular epidemiological aspects of this important pathologic condition, pointing out preventable causes and early diagnosis, will help in the implementation of targeted interventions, with a major clinical impact.

## 5. Conclusions

The sFtl-1/PIGF ratio may be an alternative method for diagnosing and classifying PE and can provide data about the severity of PE, leading to an improvement in the therapeutic management of this special pathology.

For PE, the positive limit value is over 65 pg/mL and the highest value of the sFtl-1/PIGF ratio was found in the subgroup of pregnant women with severe early-onset PE (307 pg/mL), followed by the subgroup of pregnant women with late-onset PE or GH, in which similar values were obtained (78 pg/mL, respectively 62 pg/mL). The value of the sFtl-1/PIGF ratio in the control group (normotensive pregnant women) was 3.9 pg/mL.

In conclusion, the data presented above were in accordance with existing information in previous studies, the utility of the sFtl-1/PIGF ratio being of greater importance in the diagnosis of PE before 34 weeks of gestation than in late-onset PE.

The major contribution of this study for clinical practice is that, for the first time in a national study, a limit value was established for the sFtl-1/PIGF ratio in pregnant women with PE, pregnant women with GH, and normotensive pregnancies (control group).

## Figures and Tables

**Table 1:** Clinical findings in the studied groups.

	**PE**	**GH**	**Control**	***p***
No.	59	25	43	
Maternal age (years) *	29.51 ± 6.17	31.8 ± 4.38	28.86 ± 6.45	F = 2.2004 *p* = 0.139 ^a^
Gestational age at onset (weeks) *	32.44 ± 3.11	32.40 ± 2.10	30.51 ± 3.75	F = 5.155 *p* = 0.007 ^a^

^a^ One-way ANOVA test; * median value ± standard deviation; *p* < 0.05 significant difference.

**Table 2:** Paraclinical findings in the studied groups.

		**PE**	**GH**	**Control**	***p***
No.		59	25	43	
Renal	Proteinuria (g/24 h) *	3.61 ± 2.65	0.18 ± 0.09	0.18 ± 0.14	F = 56.252 *p* < 0.001 ^a^
	Creatinine (mg/dL) *	0.81 ± 0.20	0.54 ± 0.03	0.53 ± 0.04	F = 67.022 *p* < 0.001 ^a^
Uric acid (mg/dL) *		4.99 ± 0.74	3.31 ± 0.32	3.16 ± 0.36	F = 157.02 *p* < 0.001 ^a^
Liver enzymes	ASAT (U/L) *	62.53 ± 24.43	22.24 ± 4.49	18.79 ± 3.73	F = 99.272 *p* < 0.001 ^a^
	ALAT (U/L) *	53.92 ± 16.88	13.16 ± 3.34	11.26 ± 2.84	F = 201.532 *p* < 0.001 ^a^
Platelet count (×10^4^/μL) *		18.44 ± 5.65	24.09 ± 3.58	25.86 ± 3.32	F = 35.413 *p* < 0.001 ^a^

^a^ One-way ANOVA test; * median value ± standard deviation; *p* < 0.05 significant difference.

**Table 3:** Specific values of the sFtl-1/PIGF ratio in the studied groups.

	**PE**	**GH**	**Control**	***p***
No.	59	25	43	
sFtl-1/PIGF Ratio (pg/mL) *	209 ± 138.77	46.08 ± 17.37	3.90 ± 0.20	*p* < 0.001

* median value ± standard deviation; *p* < 0.05 significant difference.

**Table 4:** Pregnant women distribution according to gestational age at onset.

Group	Gestational Age at Onset	
	Early Onset (<34 Weeks)	Late Onset (>34 Weeks)
PE (no. of pregnant women, %)	35 (59.3%)	24 (40.7%)
Gestational Hypertension (no. of pregnant women, %)	17 (68%)	8 (32%)
Control Group (no. of pregnant women, %)	32 (74.4%)	11 (25.6%)

**Table 5:** Values of the sFtl-1/PIGF ratio depending on gestational age at onset.

	PE		GH		Control	
	Early Onset	Late Onset	Early Onset	Late Onset	Early Onset	Late Onset
No.	35	24	17	8	32	11
Median	302.00	78.00	32.00	62.50	3.90	3.80
Minimum	64.00	39.00	19.00	36.00	3.50	3.60
Maximum	424.00	414.00	62.00	86.00	4.30	4.10

**Table 6:** Values of the sFtl-1/PIGF ratio (pg/mL) depending on the severity of PE.

	Degree of PE Severity	
	Mild PE	Severe PE
No.	23	36
Median	77.00	303.00
Minimum	39.00	49.00
Maximum	98.00	424.00

**Table 7:** Values of sFtl-1/PIGF ratio in PE depending on severity and onset.

	Severity of PE			
	Severe		Mild	
	Early Onset	Late Onset	Early Onset	Late Onset
No.	27	9	8	15
Med.	307.00	98.00	86.00	75.00
Min.	251.00	49.00	64.00	39.00
Max.	424.00	414.00	98.00	92.00

## Data Availability

Data used in this paper were obtained from patient files, Constanta Emergency Hospital. Any further information regarding this work is available from the corresponding author upon reasonable request.
